# Genome-Wide Identification and Analysis of the Maize Serine Peptidase S8 Family Genes in Response to Drought at Seedling Stage

**DOI:** 10.3390/plants12020369

**Published:** 2023-01-12

**Authors:** Hongwei Cui, Guyi Zhou, Hongqiang Ruan, Jun Zhao, Agula Hasi, Na Zong

**Affiliations:** 1 Biotechnology Research Institute, Chinese Academy of Agricultural Sciences, Beijing 100081, China; 2 Key Laboratory of Herbage and Endemic Crop Biology, Ministry of Education, School of Life Sciences, Inner Mongolia University, Hohhot 010020, China

**Keywords:** *Zea mays* L., *ZmSPS8s*, inbred, expression pattern, survival rate

## Abstract

Subtilisin-like proteases (subtilases) are found in almost all plant species and are involved in regulating various biotic and abiotic stresses. Although the literature on subtilases in different plant species is vast, the gene function of the serine peptidase S8 family and its maize subfamily is still unknown. Here, a bioinformatics analysis of this gene family was conducted by describing gene structure, conserved motifs, phylogenetic relationships, chromosomal distributions, gene duplications, and promoter cis-elements. In total, we identified 18 *ZmSPS8* genes in maize, distributed on 7 chromosomes, and half of them were hydrophilic. Most of these proteins were located at the cell wall and had similar secondary and tertiary structures. Prediction of cis-regulatory elements in promoters illustrated that they were mainly associated with hormones and abiotic stress. Maize inbred lines B73, Zheng58, and Qi319 were used to analyze the spatial-temporal expression patterns of *ZmSPS8* genes under drought treatment. Seedling drought results showed that Qi319 had the highest percent survival after 14 d of withholding irrigation, while B73 was the lowest. Leaf relative water content (LRWC) declined more rapidly in B73 and to lower values, and the nitrotetrazolium blue chloride (NBT) contents of leaves were higher in Qi319 than in the other inbreds. The qPCR results indicated that 6 serine peptidase S8 family genes were positively or negatively correlated with plant tolerance to drought stress. Our study provides a detailed analysis of the *ZmSPS8s* in the maize genome and finds a link between drought tolerance and the family gene expression, which was established by using different maize inbred lines.

## 1. Introduction

When plants are exposed to various environmental stimuli, endogenous phytohormones such as melatonin, polyamines (PA), and jasmonic acid (JA) levels are increased to improve the stress tolerance of plants. In addition, the plants’ cellular redox homeostasis is disrupted [1,2,3,4], while triggering an endoplasmic reticulum (ER) stress response [5]. Accumulating unfolded or misfolded proteins during ER stress results in the generation of excessive reactive oxygen species (ROS), leading to the oxidation of side chains of amino acid residues [6] and the formation of protein–protein covalent cross-linkage, which can lead to protein inactivation or denaturation [7]. If they are not rapidly degraded, oxidatively modified proteins can undergo direct fragmentation or can form large aggregates due to covalent cross-linking and increased surface hydrophobicity, which lead to cell death [8].

The serine peptidase S8 (subtilisin-like proteinases) family belongs to the subtilases (SBTs) superfamily, which is a widely distributed family of serine proteases and was first found in eukaryotes [9]. Researchers subsequently found nine subtilases in mammals that are involved in the maturation of proteins in animals [10]. It also has been reported that the subtilase gene family is ubiquitous across the plant kingdom and participates in plant developmental processes, immune responses, and abiotic stress [11,12], ranging from 56 genes in *Arabidopsis* [13,14], 63 genes in rice (*Oryza.sativa* L.) [15], 11 genes in barley (*Hordeum vulgare* L.) [16], 80 genes in grape (*Vitis vinifera*) [17], 82 genes in tomato (*Solanum lycopersicum*) [18], 23 genes in the moss (*Physcomitrella patens*) [13] to 90 in *Populus trichocarpa* [19]. As reported, there are multiple conserved domains in subtilase family proteins such as: inhibitor_I9, peptidase_S8_3, protease-associated (PA) subtilisin-like (PA_subtilisin_like), and peptidases_S8_S53 that are present in the majority of grape subtilases [17]. Different domains confer proteins with different functions, and unique combinations of domains confer proteins with a wide variety of protein functions. The I9 peptidase inhibitor domain can block the catalytic center of the protein, and thus modulate the folding and activity of the peptidase pro-enzyme [20]. The PA domain has the ability to form homodimers that can bind to the auto-inhibitory beta-hairpin domain of tomato SBT3, thus relieving the autoinhibition and generating the active enzyme. It can also determine the optimum substrate length in soybean, indicating a possible role for the PA domain in substrate selection [18,21,22]. Peptidase_S8_3 and peptidases_S8_S53 domains may have a potential role in degrading specific substrates. Interestingly, the peptidase S8 domain is always present in fungal subtilisins and is accompanied by either domain PA or inhibitor I9, but rarely with PA and I9 concurrently [23]. As the subfamily of the SBTs family, the serine peptidase S8 family members also have the same kinds of domains and appear to have independently and convergently evolved an Asp/Ser/His catalytic triad, like in the trypsin serine proteases [24]. The thermostable protease secreted by *Geobacillus collagenovorans* MO-1 was the first S8 collagenolytic protease to be studied, and can degrade collagen in the environment to produce energy from the process of converting macromolecule materials to small ones [25]. Several subtilisin-like proteases with strong collagenolytic activity are secreted by some microorganisms and may serve as targets for the development of therapeutic agents [26]; TsP (*Trichinella spiralis* peptidase), a surface and secretory protein expressed in *T. spiralisas*, as a member of the peptidase S1 family, can promote larval invasion of intestinal epithelial cells (IECs) and intestinal mucosa [27]. Moreover, plant serine peptidase S8 family members also play important roles related to regulating stress tolerance, and they may together with the 20S proteasome degrade oxidized proteins generated by environmental stress. Inactivated or denatured proteins are degraded into single amino acids or peptides for recycling by plants, which improve plant survival in a harsh environment by enhancing stress resistance [28,29].

Climate change is a new threat that could seriously aggravate irrigation water supplies and requests, and it is also a strong reason for severe drought. Drought, as one of the most widespread natural phenomena, can affect the plant life cycle and eventually result in yield loss and poor seed quality [30,31,32]. As such, excavating more drought tolerance-related genes accurately and effectively to improve plants’ drought tolerance and reduce yield losses is urgently needed to stabilize the global productivity of crops. As an economically important and globally cultivated crop, maize (*Zea mays* L.) is a staple cereal crop, accounting for 41% of the total world cereal production. About 65% of the total world maize production is used as livestock feed, 15% as human food, and the remaining 20% is mainly used for industrial purposes [33,34]. However, the growth and yield of maize were seriously influenced by drought stress, and identifying drought-responsive genes and applying them in molecular breeding is one of the effective ways to cope with drought stress.

In recent years, most of the drought tolerance genes in maize such as *DREB*, *WRKY*, *NAC*, and *bZIP* were transcription factors [31,35,36,37] and drought resistance genes encoding proteases were rarely reported in maize. The potential protease functions of serine peptidase S8 family members may contribute to the degradation of plant-inactivating and denatured proteins under drought stress, thus improving plant drought resistance. Based on this hypothesis, genome-wide identification of one gene family could help us study the unknown gene function. In our study, 18 *ZmSPS8* genes were identified in the genome, and systematic analysis was performed using bioinformatics and molecular biology methods, such as genetic structure analysis, promoter analysis, and protein structure analysis. The expression of these 18 genes in Qi319, Zheng58, and B73 seedlings at different time points before and after drought treatment and the drought tolerance of three maize inbreds were analyzed. Our findings lay the foundation for further evolutionary research on the plant serine peptidase gene family and offer some useful information for the identification of key genes in response to drought stress.

## 2. Results

### 2.1. Identification and Classification of SPS8 Proteins in Zea mays

Based on the BLASTP program and the HMM files, a total of 18 non-redundant SPS8 proteins were obtained. According to the physico-chemical characteristics predicted by the Expasy tool, we found that the protein lengths, molecular weights (MWs), and isoelectric points (pI) of the family members showed large variation. The length of the *ZmSPS8* gene’s coding region varied from 285 bp (*ZmSPS8.3.2*) to 4059 bp (*ZmSPS8.3.3*). The length of SPS8 proteins ranged from 95 aa (*ZmSPS8.3.2*) to 1352 aa (*ZmSPS8.3.3*). Molecular weight ranged from 9959.5 Da (*ZmSPS8.3.2*) to 148,109.91 Da (*ZmSPS8.3.3*). The theoretical pI of SPS8 proteins ranged from 4.84 (*ZmSPS8.1.7*) to 11.38 (*ZmSPS8.1.14*). Nine of the SPS8 proteins were hydrophilic, and the other nine proteins were hydrophobic (Figure S1). According to the subcellular localization predictions, most of the family members were localized in the cell wall, while only two proteins (ZmSPS8.2.0 and ZmSPS8.1.14) were localized in the nucleus. In addition, only one protein (ZmSPS8.3.1) was localized in the cell membrane, and ZmSPS8.1.11 was localized in both the cell wall and cell membrane. All of the molecular characteristics of the SPS8 proteins are listed in Table 1.

### 2.2. Phylogenetic Relationships Analysis, SPS8 Conserved Motifs Prediction and Gene Structure Analysis of ZmSPS8

**Figure 1:**
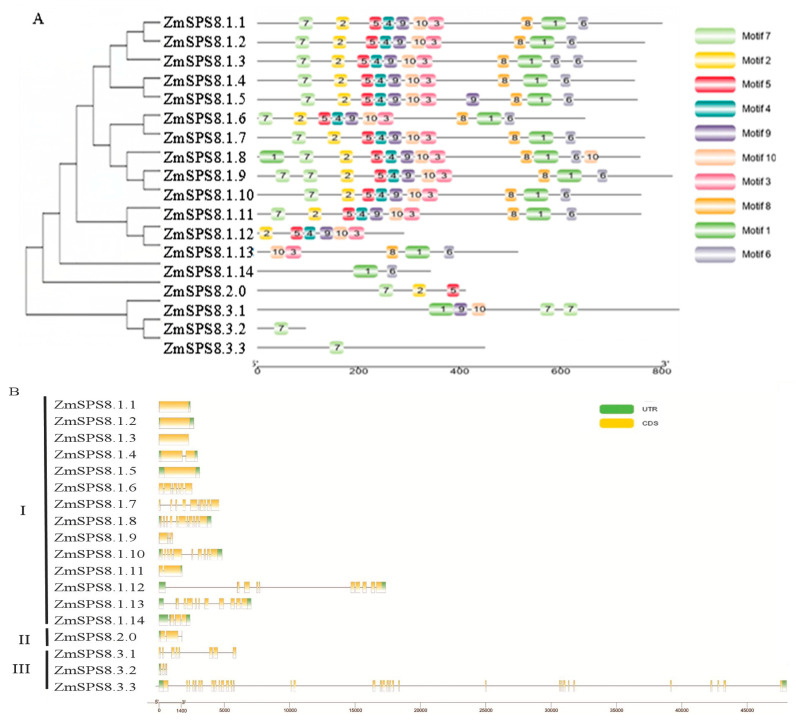
Phylogenetic relationships, conserved motifs, and gene structures of maize serine peptidase S8 family proteins. (**A**) Unrooted tree of 18 proteins and comparison of the conserved motifs of the 18 proteins. MEGA-X was used to construct the maximum-likelihood phylogenetic tree with 1000 replicates. Ten conserved motifs are shown in different colored boxes, and the details of all motifs are displayed in Supplementary File. (**B**) Exon–intron structures of 18 proteins. Yellow boxes represent exons, black lines represent introns, and the upstream/downstream regions of 18 genes are represented by blue boxes.

The full-length amino acid sequences of the 18 family members were aligned and used to construct an unrooted phylogenetic tree to analyze their phylogenetic relationships. The result showed that these family members were divided into three groups (class I–class III) with high bootstrap value support, indicating that they have a conserved phylogenetic relationship (Figure 1A). Therefore, all family members were named according to the evolutionary tree: ZmSPS8.1.1-ZmSPS8.1.14 for class I, ZmSPS8.2 for class II, ZmSPS8.3.1–ZmSPS8.3.3 for class III. The gene structure analysis was performed by TBtools software to further support their phylogenetic relationships (Figure 1B). The result revealed that the intron number in the genomic sequences of the family members ranged from 0 to 33. There is no intron in four genes (*ZmSPS8.1.5*, *ZmSPS8.1.1*, *ZmSPS8.1.3*, and *ZmSPS8.1.2*), while *ZmSPS8.3.3* contains 33 introns. Ten motifs were identified among the family members through conserved motif analysis by MEME (Figure 1A and Figure S1). These motifs were named motifs 1–10, motifs 1, 5, and 9, and were all identified as the peptidase_S8 domain (PF00082); motif 7 was the inhibitor_I9 domain (PF05922), motif 6 was the EABR domain; however, the others were unknown domains. All proteins in class I and ZmSPS8.2.1 contained all ten motifs. Except for three proteins (ZmSPS8.1.14, ZmSPS8.1.11, and ZmSPS8.1.9), other SPS8 proteins contained motif 7 which is the inhibitor I9 domain (PF05922). Combining the analysis results of the phylogenetic tree and conserved motifs, we can also find that the ZmSPS8s of the same class contained similar motif compositions, but they differed from different classes, suggesting that different classes have complementary functions, but the same class exhibits redundancy. According to the phylogenetic tree, gene structure, and conserved motif analysis, we found that the gene pair *ZmSPS8.1.1*-*ZmSPS8.1.5* exhibited highly similar conserved motifs and exon–intron organization patterns, suggesting their close relationship.

### 2.3. Chromosomal Distributions, Gene Duplications and Divergence Time

**Figure 2:**
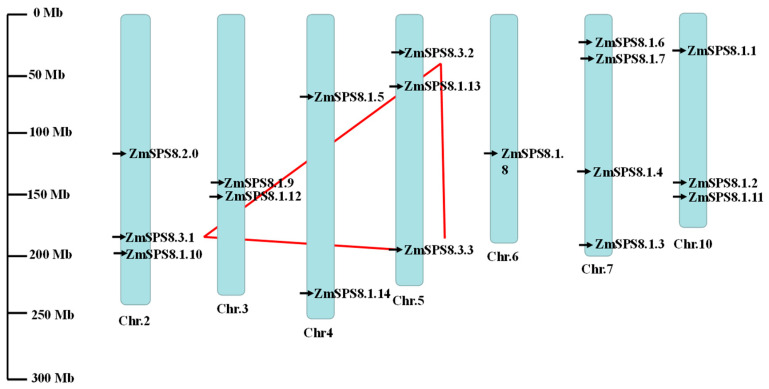
Chromosomal distribution and segmental duplication events of serine peptidase S8 family genes in maize. The duplicated paralogous pairs of family genes were connected with red dashed lines. Chromosome numbers were located under each vertical bar. Gene IDs in black represent maize serine peptidase S8 family genes.

We drew a location map of each family gene on the maize chromosomes to research the genomic distribution of the 18 genes and found that these genes are unevenly distributed on 7 of the 10 chromosomes. There are 4 genes on Chr 7; 3 genes on Chr 2, 5, and 10, respectively; 2 genes on Chr 3 and 4, respectively; only one gene on Chr 6. The chromosome location information is shown in Figure 2. Three duplicated genes were identified based on the amino acid identity >85% and gene alignment coverage >0.75, and they were divided into three groups (*ZmSPS8.3.1*/*ZmSPS8.3.2, ZmSPS8.3.1*/*ZmSPS8.3.3, ZmSPS8.3.2*/*ZmSPS8.3.3*) (Table 2). One duplicated gene pair (*ZmSPS8.3.2*/*ZmSPS8.3.3*) was distributed on the same chromosome. However, they are genetically separated on the chromosome, implying that tandem duplication events were not involved in the expansion of *ZmSPS8* genes, and all the duplicated genes showed segmental duplication. The ratios of Ka/Ks for two of these three groups were less than 1.0 (Table 2), suggesting that these two pairs had evolved mainly under purifying selection. The last pair was under positive selection because the Ka/Ks ratio was greater than 1.0 (Table 2). The divergence times of duplicated gene pairs ranged from 0.50 to 26.77 million years ago (Mya) and averaged 9.51 Mya (Table 2).

### 2.4. Prediction of the Protein Structure, Signal Peptides and Trans-Membrane Helix

**Figure 3:**
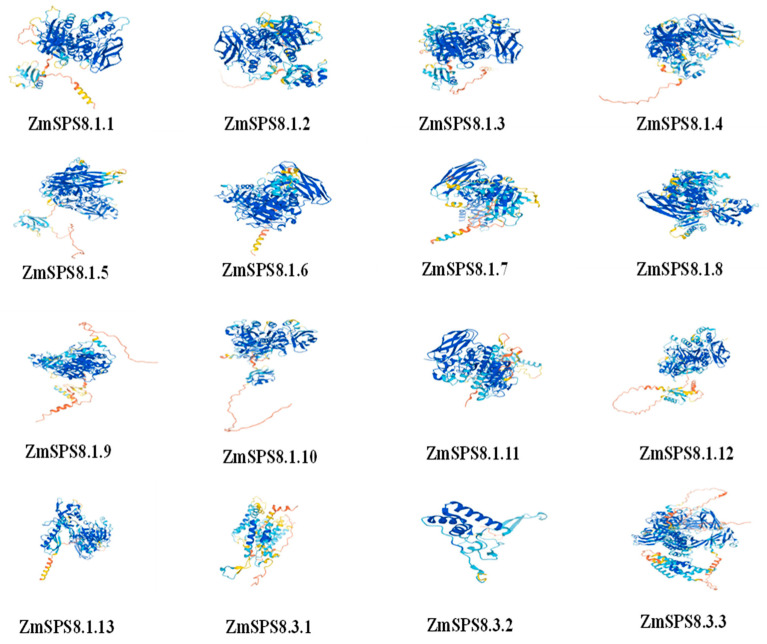
Three-dimensional Models of the serine peptidase S8 family proteins.

Secondary structure predictions revealed that the SPS8 proteins mainly consisted of alpha helices, extended strands, beta turns, and random coils (Table 3). The random coils accounted for the largest percentage of the secondary structures, followed by alpha helices and extended strands. In addition, the 3D structure models of all the proteins were predicted using the AlphaFold Protein Structure Database [38]. We found that these family proteins mostly shared similar structures, except for 2 proteins that failed prediction (Figure 3. SignalP 5.0 predicted the functional sequences of the proteins succeeding in the splitting of the signal peptide. The result showed that only 8 proteins had signal peptides. Transmembrane domain prediction with the TMHMM Server v. 2.0 discovered that there was only 1 trans-membrane helice in ZmSPS8.1.3, ZmSPS8.1.10, and ZmSPS8.1.2., while 4 trans-membrane helices in ZmSPS8.3.1.

### 2.5. Cis-Element Analysis of ZmSPS8 Genes in Maize

**Figure 4:**
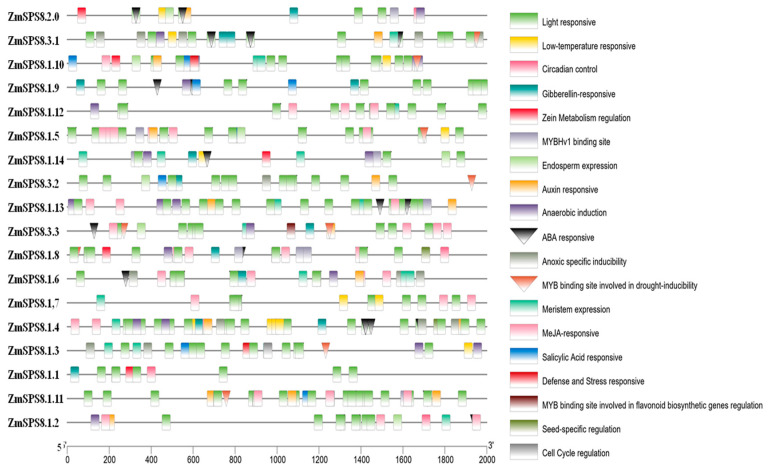
Putative regulatory cis-elements in maize serine peptidase S8 family gene promoters. Boxes of different colors represent different cis-elements and the position of drought stress-related cis-acting elements. in all genes is indicated by the inverted triangle.

Little evidence indicates that *ZmSPS8* genes play important roles in responses to abiotic stresses [39]. To explore the possibility of *ZmSPS8* genes involved in stress responses, the presence of abiotic stress-related cis-elements in 18 genes was investigated. We found that the cis-acting regulatory elements in *ZmSPS8* genes promoter regions were mainly divided into the following four categories: light, hormone, stress responsive, and growth and metabolic responsive elements (Figure 4). These stress-responsive cis-elements in promoters were involved in drought, low-temperature, defense mechanism, and anaerobic condition. In addition, cis-elements related to salicylic acid (SA), methyl jasmonate (MeJA), gibberellins (GA), and auxin (IAA) were also identified. Almost all *ZmSPS8* gene promoters contain cis-elements associated with drought stress, suggesting that the *ZmSPS8* gene family may be involved in regulating drought response.

### 2.6. Expression Patterns of ZmSPS8 Genes at Different Developmental Stages

**Figure 5:**
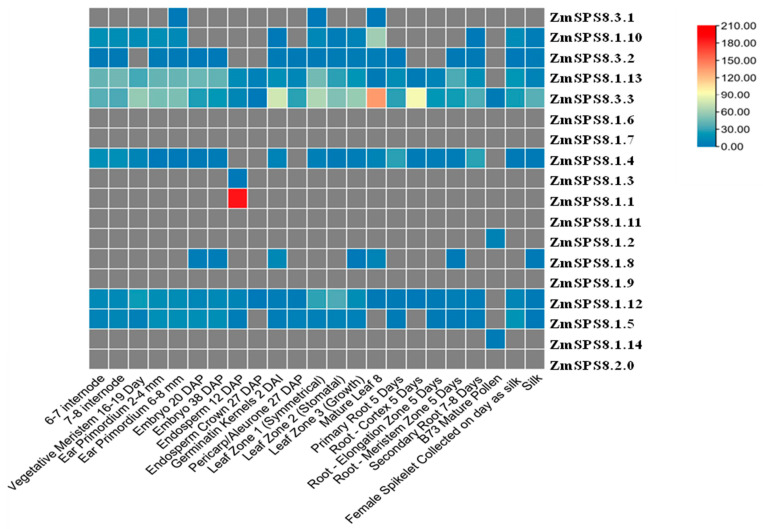
Expression pattern analysis of the maize serine peptidase S8 family genes in different tissues and organs using transcript data. Expression patterns of the 18 genes were investigated in 23 different tissues and organs using publicly transcriptome data. The fragments per kilobase of transcript per million fragments (FPKM) values of all genes were transformed by log2. The red and grey colors represent the higher and lower relative enrichment of the transcript.

To explore the possible functions of *ZmSPS8* genes, the expression patterns of the 18 family members were analyzed in 23 tissues from various development stages and organs by using publicly available transcript data [40]. Some genes showed detectable expression levels in most of the 23 tissues and developmental stages with different expression patterns, and some genes showed no expression (Figure 5). Practically, we noted that *ZmSPS8.3.3* had different degrees of expression levels in all stages. *ZmSPS8.1.3* and *ZmSPS8.1.1* only showed expression at 12 DAP (days after pollination) endosperm, while *ZmSPS8.1.2* and *ZmSPS8.1.14* only showed expression at mature pollen. Some *ZmSPS8* genes showed similar expression patterns that reflected their close relationships, especially for three pairs of genes (*ZmSPS8.1.3* and *ZmSPS8.1.1*, *ZmSPS8.1.2* and *ZmSPS8.1.14*, *ZmSPS8.1.13*, and *ZmSPS8.1.12*), which might suggest they play the same functions in plant growth and development.

### 2.7. Drought Tolerance Test of Qi319, Zheng58 and B73 Seedlings

**Figure 6:**
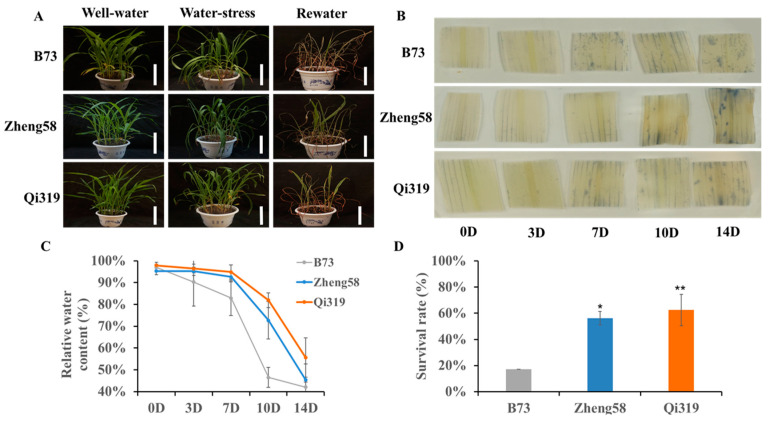
Phenotypes of three maize lines under drought treatment. (**A**) Drought treatment of B73, Zheng58, Qi319 inbred lines at different time points. (White bar = 100 mm); (**B**) ROS production and oxidative damage of three inbred lines under drought stress. NBT staining for superoxide(O_2_^−^) (blue, B) in the leaf blades; (**C**) leaf relative water content (RWC) of three maize inbred lines during two weeks of drought stress. The x-axis is the time course of treatment, and seedlings were sampled at 0(CK), 3, 7, 10, and 14 D after drought treatment, respectively. The y-axis shows the relative water content; (**D**) survival rate of drought-treated three maize inbred lines. Bars represent the mean (three or four replicates with each replicate containing 16 plants) ± standard deviation. The x-axis is B73, Zheng58, Qi319 inbred line and the y-axis shows the survival rate of three inbred lines. *p*-value of <0.05 (*), <0.01 (**).

We conducted a drought tolerance test on seedlings of three maize inbred lines Qi319, Zheng58, and B73. These three maize lines all grew normally under well-watered conditions, and drought stress was imposed by continuously withholding water from soil-grown plants. As shown in Figure 6, under well-watered conditions, the growth of all three inbred plants was similar and healthy, while after 7 days of drought stress treatment (the soil relative water content (SRWC) 9.0%.), all leaves of the B73 severely curled and wilted, the leaves of Zheng58 began to curl down from the base of the blade, but Qi319 showed still vigorously. After 10 days of drought treatment (SRWC 8.1%.), Zheng58 leaves were too severely withered to stand up, the Qi319 leaves also showed withered and drooped, while the whole plants of the B73 showed totally withered. All leaves of the three inbred lines curled and wilted seriously after 14 days of drought treatment (Figure 6A). The SR (survival rate) of plants was measured 7 days after re-watering; the survival rates of Qi319, Zheng58, and B73 were 62.5%, 56.25%, and 17.18%, respectively, and those of well-watered plants were 100% (Figure 6D). The morphological changes of the plants with increasing drought treatment time are shown in Supplementary Figure S2.

Leaf RWC (relative water content) of the tested plants was assayed. The leaf RWC of all plants decreased to varying degrees with the imposition of drought stress. As shown in Figure 6C, the leaf RWC of B73 decreased by 36.4% after 10 days of drought treatment, and Qi319 and Zheng58 decreased by 12.1% and 19.9%, respectively. The result indicated that the water-retaining capacity of B73 was weaker than Qi319 and Zheng58. Drought stress has been reported to accelerate the accumulation of ROS, and the overproduction of ROS in plants can cause chlorosis and cell death. To investigate whether chlorotic and cell death were observed at the adaxial side of the tested plants, the superoxide radicals of the tested leaves were stained by NBT. Our results showed that B73 accumulated more O_2_^·−^ than Qi319 and Zheng58 at 7 days, 10 days, and 14 days after drought treatment (Figure 6B).

### 2.8. Expression Analysis of ZmSPS8 Genes under Drought Treatment

**Figure 7:**
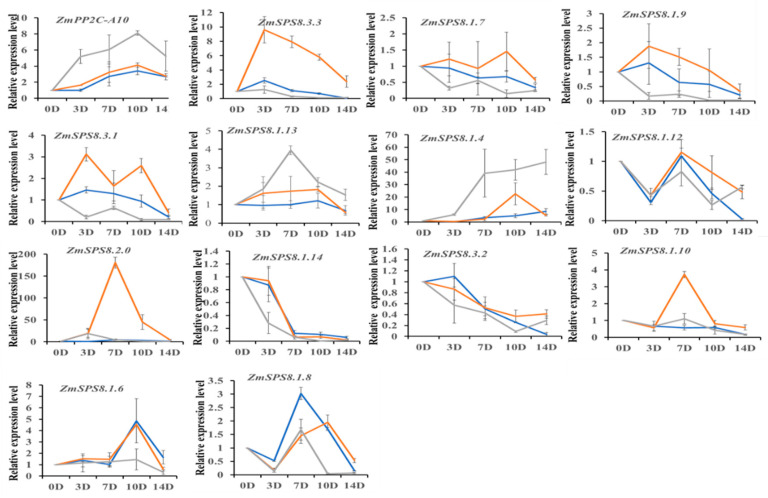
Expression pattern analysis of the maize serine peptidase S8 family genes under drought treatment by qRT-PCR. The x-axis is the time course of treatment, and seedlings were sampled at 0 (CK), 3, 7, 10, and 14 D after drought treatment, respectively. The y-axis shows the relative expression levels. Grey line: B73; Bule line: Zheng58; Orange line: Qi319.

In order to analyze if SPS8 proteins were involved in drought tolerance regulation, we used qRT-PCR to detect the expression of the *ZmSPS8* genes in the three inbred lines before and after drought treatment. As shown in Figure 7, the expression of *ZmSPS8.3.3*, *ZmSPS8.3.1*, *ZmSPS8.1.7*, and *ZmSPS8.1.9* in the three maize lines was increased or decreased compared to that of untreated, but their expression levels varied among the three maize inbred lines; the highest expression was in Qi319, followed by Zheng58, and the lowest level of expression was in B73. However, exactly the opposite was observed in *ZmSPS8.1.13* and *ZmSPS8.1.4*: the highest expression levels of them were in B73, and the lowest expression was in Qi319. In addition, expression levels of *ZmSPS8.1.14* and *ZmSPS8.3.2* were significantly down-regulated throughout the experiment time, and those of *ZmSPS8.2.0* and *ZmSPS8.1.10* were significantly up-regulated only in Qi319. Notably, some genes were differentially expressed at only a single time point. For example, *ZmSPS8.1.6* expression was significantly up-regulated at 10 days, but significantly down-regulated expression was observed at 14 days in Qi319 and Zheng58. *ZmSPS8.1.8* expression was significantly up-regulated at 7 days in all inbred lines, but this gene could still remain up-regulated only in Qi319 from 7 to 10 days after drought treatment. The remaining five genes among the family members did not have detectable expression, indicating that they might be pseudogenes or are not expressed in the leaves at the maize seedling stage. These findings suggested the important roles of *ZmSPS8* genes in response to drought stress, but they may have different regulatory mechanisms (Figure 7).

## 3. Discussion

Plant subtilases are a very diverse and widely distributed subtilisin-like family of serine proteases. They have been reported to be involved in the breakdown and replacement of proteins or protein complexes, protein post-translational processing, plant reproductive organ development, physiological modification of cell walls, abiotic and biotic responses, and apoptosis [9,19,20,21]. Plants use escape strategies, including premature senescence and leaf reduction, to cope in response to abiotic stresses such as drought and heat. The current study found that chloroplast degradation and proteolysis are implicated in the senescence processes of plants, and genes involved in these biological processes show increased expression. This suggests that proteases play crucial roles in abiotic stress responses in plants. [41,42,43]. To date, however, there have been few reports on the potential biological function of the serine peptidase S8 subfamily in maize. In this article, we identified and characterized 18 maize *SPS8* family genes and speculated on their potential effect on drought.

These 18 *SPS8* family genes showed different chromosome distribution and the coded proteins showed various pI, stability, and subcellular location prediction. The prediction results showed that most of the serine peptidase S8 family members localized to the cell wall, and a few were predicted to localize to the cell membrane and nucleus. It implies that they may be involved in various cell wall-related physiological and biochemical processes. It is reported that plants invest available resources into root growth to explore residual water in the soil and reduce shoot growth when exposed to drought stress [44], and that process is accompanied by cell wall synthesis and remodeling [45]. Another study reported some cell wall synthesis-related genes in soybean leaf under drought and flooding conditions using RNA-seq, and these genes were up-regulated under drought stress and down-regulated under flooding stress, which indicated that cell wall modification may be a protective strategy against drought stress [46]. Laura et al. [47] found that the cell wall integrity sensor THESEUS1 modulates the mechanical properties of walls, turgor loss point, ABA biosynthesis (ABA is essential for plant adaptation to drought stress and has therefore been investigated extensively in guard cells), and ABA-controlled processes in *Arabidopsis thaliana*, which further showed that responses to drought depend on the presence of a functional cell wall. A tissue-specific NAC gene called necrotic upper tips1 (*nut1*) was associated with secondary cell wall formation in the protoxylem. When this gene was lost, protoxylem vessels became thinner, resulting in the defective formation of secondary cell walls. Ultimately, it led to a block in water transport and reduced drought tolerance [48]. Xingming et al. [49] studied a rice *drought*-tolerant gene *DROUGHT1*(*DROT1*), which is specifically expressed in vascular bundles. Its protein is primarily located on the periphery of the cell, especially on the cell wall, and may specifically promote cellulose synthesis under drought stress to protect cell wall integrity. Over-expression of *DROT1* in rice plants showed improved drought tolerance, and when knocked out, the plants exhibited significantly reduced drought resistance.

Cis-elements in gene promoters are important for transcriptional regulation. For our promoter analysis result, a series of binding sites of transcription factors related to plant growth and development were identified in the promoters of these 18 genes. For example, light-responsive elements presented in the promoters of all these genes, stress-responsive cis-elements, including ABA-responsive (ABRE), drought-responsive (MBS), and low temperature-responsive (LTRE) were also found in the promoters of these 18 genes. Almost all family members contain MBS or ABRE, or both, suggesting the important roles of 18 genes in drought stress responses.

Abiotic stress such as drought reduces plant growth and survival. In our study, three maize inbred lines (Qi319, Zheng58, and B73) were selected. Drought stress for 7 days exhibited deleterious effects on B73 which caused significant leaf curling and wilted with 9% SRWC. While SRWC dropped to 8.1%, Qi319 leaves started to curl, and Zheng58 already showed severe leaf curling. In addition, plenty of studies have also used plant SR to evaluate drought tolerance: Overexpression of *ZmDREB2.7* in Arabidopsis resulted in rising survival rates through acquired drought treatment [35] and the transgenic maize with enhanced *ZmVPP1* gene expression showed an increased survival rate relative to the B73 inbred line (CK) under water-deficit conditions [40]. Interestingly enough, the transgenic plants of the negative regulator of drought tolerance GhWRKY33, OsJAZ1, or AtPUB19 reduced their SR compared with the wild type (WT) [50,51,52]. In addition to SR, leaf RWC is also positively correlated with drought resistance ability and can truly reflect the degree of water deficit in plants under drought stress, the higher Leaf RWC, the stronger the drought tolerance, and conversely, the weaker the drought tolerance [53]. According to the results of Leaf RWC, LRWC declined more rapidly in B73, such that it was 45% at 10 d, and 42% at 14 d, whereas it remained higher in Zheng58 and Qi319, such that in Qi319 LRWC was 55% at 14 d (Figure 6C). Thus, we conclude that B73 is a drought-sensitive line, which is in good agreement with other literature reports [36,40]. Likewise, histochemical staining with NBT also indicated that the ROS-scavenging capacity of B73 is weaker than that of Zheng58 and Qi319. Taken together, we conclude that Qi319 is the most tolerant inbred, followed by Zheng58, and B73.

It has been documented that drought-tolerant inbred lines, such as CIMBL55, 92, 70, and CML118, have significantly higher expression of *ZmNAC111* than drought-sensitive inbred lines (B73, Mo17) with lower expression of *ZmNAC111* [36]. In our research, we found the spatial-temporal expression of four members of the serine peptidase S8 gene family (*ZmSPS8.3.3*, *ZmSPS8.3.1*, *ZmSPS8.1.9*, *ZmSPS8.1.7*) was consistent with *ZmNAC111* gene expression results. This finding indicated that these genes expression positively correlated with maize drought tolerance. Moreover, another gene negatively associated with maize drought tolerance was *ZmPP2C-A10*, which was used as a reference gene in our study. The expression of *ZmPP2C-A10* was up-regulated in both drought-sensitive and drought-tolerant inbreds, but the up-regulated levels of this gene were significantly different, with higher levels of *ZmPP2C-A10* in sensitive inbreds than those in tolerant ones [54]. The expression of *ZmSPS8.1.13* and *ZmSPS8.1.4* was consistent with ZmPP2C-A10 gene expression, suggesting these two genes negatively correlated with maize drought tolerance. Finally, based on the published RNA-seq data, we found that part of the S8 gene family genes was not expressed in all of the 23 tissues and organs including *ZmSPS8.1.6*, *ZmSPS8.1.7*, *ZmSPS8.1.9*, *ZmSPS8.1.11*, and *ZmSPS8.2.0* while four of them were demonstrated to have significant changes in the expression levels under drought stress in qRT-PCR analysis results. The possible reasons are essentially two-fold: one is that part of the genes is drought induced, so they are upregulated; the other is that the inbred line used for the public data was B73, and the expression of genes may vary in different inbred lines.

## 4. Conclusions

The maize serine peptidase S8 family was identified and characterized in our study. Eighteen genes were obtained, and among them, five members were involved in segmental duplication. Phylogenetic relationship analysis clearly divided the family members into three groups. These 18 genes were all distributed on 7 chromosomes, and half of them were hydrophilic Most of these subtilases were located on the cell wall and had similar secondary and tertiary structures. Prediction of cis-regulatory elements in promoters illustrated that they were mainly associated with hormones and abiotic stress responses. The drought tolerance of B73, Zheng58, and Qi319 was tested at the seedling stage. The results suggested that Qi319 was the most tolerant inbred, and we also found that *ZmSPS8.3.3*, *ZmSPS8.3.1*, *ZmSPS8.1.9*, and *ZmSPS8.1.7* were positively correlated with maize drought tolerance, while *ZmSPS8.1.13* and *ZmSPS8.1.4* were negatively correlated with maize drought tolerance. Our study first links the changes in serine peptidase S8 family members’ expression under drought treatment with the strength of drought tolerance in maize inbred lines, which could provide a scientific foundation for a comprehensive understanding of the maize serine peptidase S8 family and provide new clues to drought tolerance breeding.

## 5. Materials and Methods

### 5.1. Identification and Characterization of ZmSPS8 Genes in Maize

To identify peptidase-encoding proteins in the maize genome, we downloaded genome sequences of maize from the MaizeGDB database (https://www.maizegdb.org/) (accessed on 1 December 2021). Hidden Markov model (HMM) profiles of the Peptidase_S8 domain (PF00082) were initially obtained from the Pfam database (http://pfam.xfam.org/) (https://www.maizegdb.org/) (accessed on 3 December 2021) [55] and were used for HMMSEARCH in the maize proteome with an E-value equal to 1 × 10^−10^. We searched maize SPS8 proteins using the Basic Local Alignment Search Tool Algorithm program (BLASTP), with the published *Zea mays* peptidase protein sequences used as query sequences. BLASTP is based on the protein homology alignment with an E-value equal to 1 × 10^−10^. To validate the accuracy of these candidate genes, we applied Web CD-Search Tools and SMART to confirm the domain in maize SPS8 proteins. After removing the redundant sequences, 18 members were used for further analysis. The ExPASy (https://web.expasy.org/protparam/) (3 December 2021) and Plant-mPLoc online tools (Plant-mPLoc server (sjtu.edu.cn)) (3 December 2021) were used to calculate physico-chemical characteristics including molecular weight, isoelectric point, instability index, aliphatic index, grand average of hydropathicity, etc.

### 5.2. Phylogenetic Analysis of SPS8 Proteins

To analyze the evolutionary relationships of maize SPS8 proteins, the full-length amino acid sequences of these 18 proteins from maize were used to construct a phylogenetic tree with the MEGA-X (Molecular Evolutionary Genetics Analysis) software. All the sequences were obtained from PLAZA 4.0 (https://bioinformatics.psb.ugent.be/plaza/versi-ons/plaza_v4_monocots/) (accessed on 5 December 2021). The phylogenetic tree was built using the maximum likelihood method and the Jones–Taylor–Thorton (JTT) model with 1000 bootstrap replicates.

### 5.3. Conserved Motif, Gene Structure and Promoter Analysis of ZmSPS8 Genes in Maize

Multiple Em for Motif Elicitation (MEME Version 5.4.1) (https://meme-suite.org/meme/tools/meme) (accessed on 6 December 2021) was used to predict conserved motifs in 18 amino acid sequences of ZmSPS8s [56]. The number of motifs was set to 10, and with motif widths constrained to between 6 and 50 residues. Using the Pfam database, the detected motifs were annotated. The gene structure was analyzed by Gene Structure Display Server (GSDS 2.0) (https://mybiosoftware.com/gsds-2-0-gene-structure-display-server.html) (7 December 2021) [57]. Briefly, 2000 bp flanking sequences upstream of the transcription start site (ATG) of the ZmSPS8 genes were extracted from the maize genomic sequence, and these promoters were used to predict cis-acting by PlantCARE (http://bioinformatics.psb.ugent.be/webtools/plantcare/html/) (accessed on 8 December 2021) [58,59].

### 5.4. Distribution of ZmSPS8 Genes on Chromosomes and Their Duplications and Divergence Time

Maize genome annotation information was obtained from the gff3 file (*Zea mays*. Zm-B73-REFERENCE-NAM-5.0.51.gff3), including gene location and gene structure. The *ZmSPS8* genes’ location was displayed on corresponding maize chromosomes by the TBtools software v0.667 (https://github.com/CJ-Chen/TBtools) (accessed on 9 December 2021). To confirm the gene duplications, 18 CDS sequences were blast searched against each other by using blastn of NCBI. At least 85% amino acid identity and 75% gene alignment coverage were needed (E-value < 1 × 10^−10^). To reckon the divergence of homologous genes and the selective pressure against the duplicated genes, TBtools software (v0.667) was applied to calculate the Ks (synonymous) and Ka (nonsynonymous) replacement rate per site between the members of each gene pair. Ks values > 2.0 must be discarded to avoid the risk of substitution saturation. The divergence times (T) of the gene pairs were estimated by the formula: T = Ks/2λ, with the divergence rate λ = 1.5 × 10^−8^.

### 5.5. Signal Peptides and Trans-Membrane Helix Analysis of SPS8 Proteins

SignalP-5.0 was used to predict secretion signals for SPS8 proteins (https://services.h-ealthtech.dtu.dk/service.php?SignalP-5.0) (accessed on 9 December 2021), and TMHMM Server v.2.0 was used to analyze whether trans-membrane helices existed in SPS8 proteins (https://www.hsls.pitt.e-du/obrc/index.php?page=URL1164644151) (accessed on 10 December 2021).

### 5.6. Secondary Structure Prediction and 3D Model Construction of SPS8 Proteins

Secondary structure prediction was performed using the SOPMA secondary structure prediction method (https://npsa-prabi.ibcp.fr/cgi-bin/npsa_automat.pl?page=npsa_sopma.html) (accessed on 11 December 2021). Furthermore, 3D models of peptidase proteins based on protein homology modeling were constructed by AlphaFold Protein Structure Database (https://www.alpha-fold.ebi.ac.uk/) (accessed on 11 December 2021) [38].

### 5.7. Expression Patterns of ZmSPS8 Genes in Different Tissues and Organs

To explore the expression patterns of *ZmSPS8* genes in maize, the publicly available transcriptome data published by Walley et. al. [60] 23 different developmental stages were collected from MaizeGDB (https://www.maizegdb.org/) (accessed on 11 December 2021). Gene expression levels were expressed as fragments per kilobase of transcript per million fragments mapped (FPKM). We visualized the expression data using standard heat maps.

### 5.8. Plant Materials and Stress Treatments

Maize inbred lines Qi319, Zheng58, and B73 were selected for this study. All three of these inbred lines are parents of a high-yielding hybrid and are frequently used in maize genetics research. Seeds of inbred lines were surface-sterilized, washed with sterile water three times, and germinated in vermiculites until the coleoptile grew to about 2 cm in length. The seeds with consistent germination were selected. Twenty seeds per pot (pot measuring 23 cm in diameter and 17 cm in height) were planted in the greenhouse, each pot was filled with 1 kg of soil, and 16 seedlings were retained one week after seeding emergence. Seedlings were well watered until they reached the three-leaf stage, then drought stress was imposed by withholding water, except for control plants that were watered as usual. Three replicates were set for two treatments: well-watered (WW) and water-stressed (WS). After two weeks of drought stress, all the drought-stressed plants were re-watered. Final survival was analyzed 7 days after rewatering (survival rate calculation formula: survival rate (SR%) = survival quantity/total quantity × 100%). At days 0, 3, 7, 10, and 14 after drought treatment, the third true leaf of each seedling (three leaves from each replicate,) was harvested and flash frozen by liquid nitrogen and stored at −80 °C before RNA isolation. To measure leaf relative water content (RWC), three leaves were removed from each replicate, the 7 cm mid-section was cut from each leaf, and the fresh weight (FW) was measured. This excised section was then placed in a sterile tube containing 10 mL tap water, capped, and left at 25 °C for 12 h. After this time, the leaf sections were blotted carefully, and turgid weight (TW) was measured. The sections were then dried for 12 h at 80 °C in the drying oven for the dry weight (DW). RWC (%) was calculated as (FW-DW/TW-DW) 100%.

### 5.9. RNA Isolation and Quantitative Real-Time PCR (qRT-PCR) Analysis

Total RNA was extracted using Trizol reagent (Mona, China). First-strand cDNA was synthesized by the HiScript III All-in-one RT SuperMix Perfect for qPCR(Vazyme, Nanjing, China). 2 × RealStar Green Fast Mixture with ROX II (GenStar, Fuzhou, China) was used for real-time fluorescence quantitative analysis. Fourteen specific primers for *ZmSPS8* genes were synthesized at the Tsingke Biotechnology Company (Beijing, China) (Table S1). The PCR program was 2 min at 95 °C, followed by 40 cycles of 15 s at 95 °C, 30 s at 60 °C, and 30 s at 72 °C. The specificity of the reactions was confirmed by the machine standard melt curve method. *β-actin* served as an internal control.

### 5.10. NBT Staining Assay

We used histochemical staining to detect superoxide in situ in accordance with a previously published report [39,47]. Leaf segments of 2 cm were cut from the middle part of the third leaves of maize plants at days 0, 3, 7, 10, and 14 after drought treatment (the third true fully expanded leaf of each seedling was used, three replicates were used). All the leaves were vacuum infiltrated with NBT solution (0.5 mg/mL) in the dark for 2 h at 25 °C. Then, the leaves were boiled in 100% ethanol for about 30 min to remove the chlorophyll before imaging. Seedlings that grew under normal water conditions were used as controls.

### 5.11. Statistical Analysis

Data were analyzed using SPSS v22.0 (SPSS, Chicago, IL, USA), and a one-way ANOVA was used to assess the significance of the experimental results. All data are presented as the means ± standard error of the mean. Differences were considered significant at a *p*-value of <0.05 (*), <0.01 (**), or <0.001 (***).

## Figures and Tables

**Table 1:** Characterization of the serine peptidase S8 family genes and its proteins.

Gene Name	Sequence ID	Chr	Gene Length (bp)	Number of Amino Acid (aa)	Theoretical pI	Molecular Weight (Da)	Instability Index	Aliphatic Index	Grand Average of Hydropathicity	Subcellular Predicted
*ZmSPS8.1.3*	Zm00001eb331110	Chr7	2262	753	7.11	77,699.79	31.87	85.92	0.054	Cell wall.
*ZmSPS8.1.9*	Zm00001eb150910	Chr3	873	290	8.31	30,112.91	29.16	87.17	0.108	Cell wall.
*ZmSPS8.1.10*	Zm00001eb112750	Chr2	2277	758	7.28	80,160.82	38.76	85.95	−0.007	Cell wall.
*ZmSPS8.1.4*	Zm00001eb314120	Chr7	2301	766	8.23	79,529.89	39.87	82.99	0.009	Cell wall.
*ZmSPS8.1.5*	Zm00001eb177190	Chr4	2409	802	8.22	81,600.43	38.52	85.51	0.15	Cell wall.
*ZmSPS8.2.0*	Zm00001eb095400	Chr2	1236	411	8.58	43,522.29	51.42	83.43	−0.026	Nucleus.
*ZmSPS8.1.14*	Zm00001eb205880	Chr4	1029	342	11.38	36,610.6	69.11	70	−0.461	Nucleus.
*ZmSPS8.1.12*	Zm00001eb152020	Chr3	2283	760	6.00	79,871.07	28.59	85.37	0.074	Cell wall.
*ZmSPS8.1.6*	Zm00001eb302430	Chr7	1947	648	8.93	67,846	41.41	83.01	0.024	Cell wall.
*ZmSPS8.1.8*	Zm00001eb275670	Chr6	2304	767	9.40	81,577.47	43.31	74.68	−0.153	Cell wall.
*ZmSPS8.1.1*	Zm00001eb408980	Chr10	2241	746	5.63	78,339.51	38.56	84.34	−0.039	Cell wall.
*ZmSPS8.1.13*	Zm00001eb226820	Chr5	2466	821	7.35	87,158.25	27.41	85.91	−0.012	Cell wall.
*ZmSPS8.3.2*	Zm00001eb223440	Chr5	285	94	9.67	9959.5	43.06	87.23	−0.137	Cell wall.
*ZmSPS8.3.1*	Zm00001eb111040	Chr2	1551	516	8.93	47,421.61	41.09	94.45	0.324	Cell membrane
*ZmSPS8.1.11*	Zm00001eb419540	Chr10	1350	449	5.57	53,920.23	39.6	92.87	0.239	Cell membrane/Cell wall.
*ZmSPS8.1.2*	Zm00001eb419570	Chr10	2280	759	6.25	79,388.55	27.25	88.05	0.106	Cell wall.
*ZmSPS8.1.7*	Zm00001eb302440	Chr7	2256	751	4.84	77,779.66	36.47	77.62	−0.055	Cell wall.
*ZmSPS8.3.3*	Zm00001eb249900	Chr5	4059	1352	5.95	148,109.91	39.01	89.07	−0.238	Cell wall.

**Table 2:** Inference of duplication time in paralogous pairs.

Paralogous Pairs	Gene Alignment Coverage	Ka	Ks	Ka/Ks	Divergence Time (MYA)
*ZmSPS8.3.2/ZmSPS8.3.3*	0.993	0.004817359	0.014907323	0.32315386	0.496910769
*ZmSPS8.3.1/ZmSPS8.3.2*	0.957	0.062432033	0.038079244	1.639529208	1.269308134
*ZmSPS8.3.3/ZmSPS8.3.1*	0.966	0.558916065	0.803034338	0.696005187	26.76781128

**Table 3:** Secondary structural statistics of the serine peptidase S8 family proteins.

Gene Name	Alpha Helix (%)	Extended Strand (%)	Beta Turn (%)	Random Coil (%)
*ZmSPS8.1.3*	16.73	25.37	6.64	51.26
*ZmSPS8.1.9*	16.90	25.52	8.96	48.62
*ZmSPS8.1.10*	17.81	24.93	5.15	52.11
*ZmSPS8.1.4*	19.58	25.20	6.79	48.43
*ZmSPS8.1.5*	17.08	23.45	6.98	52.49
*ZmSPS8.2.0*	41.36	13.38	4.87	40.39
*ZmSPS8.1.14*	23.10	11.99	8.77	56.14
*ZmSPS8.1.12*	20.00	21.97	6.45	51.58
*ZmSPS8.1.6*	16.51	26.54	5.72	51.23
*ZmSPS8.1.8*	19.03	23.99	6.39	50.59
*ZmSPS8.1.1*	18.63	23.59	6.84	50.94
*ZmSPS8.1.13*	22.41	20.58	5.60	51.41
*ZmSPS8.3.2*	37.23	13.83	5.32	43.62
*ZmSPS8.1.11*	16.86	25.19	5.04	52.91
*ZmSPS8.3.1*	25.84	25.39	7.35	41.42
*ZmSPS8.1.2*	20.42	23.72	6.19	49.67
*ZmSPS8.1.7*	18.77	23.17	6.39	51.67
*ZmSPS8.3.3*	31.58	20.56	5.25	42.61

## Data Availability

Data available on request from the authors.

## Supplementary Materials

The following supporting information can be downloaded at: https://www.mdpi.com/article/10.3390/plants12020369/s1, Figure S1: Schematic of ZmSPS8 conserved motifs. Ten conserved motifs were identified using MEME; Figure S2: The morphological changes of three maize lines with increasing drought treatment time; Table S1: Primer sequences for qRT-PCR analysis.

## References

[1] Suzuki H., Yokawa K., Nakano S., Yoshida Y., Fabrissin I., Okamoto T., Baluška F., Koshiba T. (2016). Root cap-dependent gravitropic U-turn of maize root requires light-induced auxin biosynthesis via the YUC pathway in the root apex. J. Exp. Bot..

[2] Borchers A., Pieler T. (2010). Programming pluripotent precursor cells derived from Xenopus embryos to generate specific tissues and organs. Genes.

[3] Gao C., Sheteiwy M.S., Han J., Dong Z., Pan R., Guan Y., Alhaj Hamoud Y., Hu J. (2020). Polyamine biosynthetic pathways and their relation with the cold tolerance of maize (*Zea mays* L.) seedlings. Plant Signal. Behavoir.

[4] Sheteiwy M.S., Ulhassan Z., Qi W., Lu H., AbdElgawad H., Minkina T., Sushkova S., Rajput V.D., El-Keblawy A., Josko I. (2022). Association of jasmonic acid priming with multiple defense mechanisms in wheat plants under high salt stress. Front. Plant Sci..

[5] He Z., Wu J., Sun X., Dai M. (2019). The maize clade A PP2C phosphatases play critical roles in multiple abiotic stress responses. Int. J. Mol. Sci..

[6] Dudziak K., Bulak P., Zapalska M., Börner A., Szczerba H., Leśniowska-Nowak J., Nowak M. (2019). Using intervarietal substitution lines for the identification of wheat chromosomes involved in early responses to water-deficit stress. PLoS ONE.

[7] Janiak A., Kwasniewski M., Sowa M., Gajek K., Żmuda K., Kościelniak J., Szarejko I. (2018). No time to waste: Transcriptome study reveals that drought tolerance in barley may be attributed to stressed-like expression patterns that exist before the occurrence of stress. Front. Plant Sci..

[8] Magwanga R.O., Kirungu J.N., Lu P., Cai X., Zhou Z., Xu Y., Hou Y., Agong S.G., Wang K., Liu F. (2019). Map-based functional analysis of the *GhNLP* genes reveals their roles in enhancing tolerance to N-deficiency in cotton. Int. J. Mol. Sci..

[9] Xu Y., Wang S., Li L., Sahu S.K., Petersen M., Liu X., Melkonian M., Zhang G., Liu H. (2019). Molecular evidence for origin, diversification and ancient gene duplication of plant subtilases (SBTs). Sci. Rep..

[10] Seidah N.G., Chrétien M. (1999). Proprotein and prohormone convertases: A family of subtilases generating diverse bioactive polypeptides. Brain Res..

[11] Ran L.-Y., Su H.-N., Zhou M.-Y., Wang L., Chen X.-L., Xie B.-B., Song X.-Y., Shi M., Qin Q.-L., Pang X. (2014). Characterization of a novel subtilisin-like protease myroicolsin from deep sea bacterium Myroides profundi D25 and molecular insight into its collagenolytic mechanism. J. Biol. Chem..

[12] Feng J., Hwang R., Hwang S.F., Strelkov S.E., Gossen B.D., Zhou Q.X., Peng G. (2010). Molecular characterization of a serine protease Pro1 from *Plasmodiophora brassicae* that stimulates resting spore germination. Mol. Plant Pathol..

[13] Rautengarten C., Steinhauser D., Büssis D., Stintzi A., Schaller A., Kopka J., Altmann T. (2005). Inferring hypotheses on functional relationships of genes: Analysis of the *Arabidopsis thaliana* subtilase gene family. PLoS Comput. Biol..

[14] Beers E.P., Jones A.M., Dickerman A.W. (2004). The S8 serine, C1A cysteine and A1 aspartic protease families in *Arabidopsis*. Phytochemistry.

[15] Tripathi L.P., Sowdhamini R. (2006). Cross genome comparisons of serine proteases in *Arabidopsis* and rice. BMC Genom..

[16] Roberts I.N., Veliz C.G., Criado M.V., Signorini A., Simonetti E., Caputo C. (2017). Identification and expression analysis of 11 subtilase genes during natural and induced senescence of barley plants. J. Plant Physiol..

[17] Cao J., Han X., Zhang T., Yang Y., Huang J., Hu X. (2014). Genome-wide and molecular evolution analysis of the subtilase gene family in *Vitis vinifera*. BMC Genom..

[18] Reichardt S., Repper D., Tuzhikov A.I., Galiullina R.A., Planas-Marquès M., Chichkova N.V., Vartapetian A.B., Stintzi A., Schaller A. (2018). The tomato subtilase family includes several cell death-related proteinases with caspase specificity. Sci. Rep..

[19] Schaller A., Stintzi A., Graff L. (2012). Subtilases–versatile tools for protein turnover, plant development, and interactions with the environment. Physiol. Plant..

[20] Figueiredo J., Sousa Silva M., Figueiredo A. (2018). Subtilisin-like proteases in plant defence: The past, the present and beyond. Mol. Plant Pathol..

[21] Rose R., Schaller A., Ottmann C. (2010). Structural features of plant subtilases. Plant Signal. Behav..

[22] Tanaka H., Onouchi H., Kondo M., Hara-Nishimura I., Nishimura M., Machida C., Machida Y. (2001). A subtilisin-like serine protease is required for epidermal surface formation in *Arabidopsis* embryos and juvenile plants. Development.

[23] Armijos Jaramillo V.D., Vargas W.A., Sukno S.A., Thon M.R. (2013). Horizontal transfer of a subtilisin gene from plants into an ancestor of the plant pathogenic fungal genus Colletotrichum. PLoS ONE.

[24] Höllbacher B., Schmitt A.O., Hofer H., Ferreira F., Lackner P. (2017). Identification of proteases and protease inhibitors in allergenic and non-allergenic pollen. Int. J. Mol. Sci..

[25] Itoi Y., Horinaka M., Tsujimoto Y., Matsui H., Watanabe K. (2006). Characteristic features in the structure and collagen-binding ability of a thermophilic collagenolytic protease from the thermophile Geobacillus collagenovorans MO-1. J. Bacteriol..

[26] Bastianelli G., Bouillon A., Nguyen C., Le-Nguyen D., Nilges M., Barale J.-C. (2014). Computational design of protein-based inhibitors of Plasmodium vivax subtilisin-like 1 protease. PLoS ONE.

[27] Lei J.J., Hu Y.Y., Liu F., Yan S.W., Liu R.D., Long S.R., Jiang P., Cui J., Wang Z.Q. (2020). Molecular cloning and characterization of a novel peptidase from *Trichinella spiralis* and protective immunity elicited by the peptidase in BALB/c mice. Vet. Res..

[28] Book A.J., Yang P., Scalf M., Smith L.M., Vierstra R.D. (2005). Tripeptidyl peptidase II. An oligomeric protease complex from *Arabidopsis*. Plant Physiol..

[29] Polge C., Jaquinod M., Holzer F., Bourguignon J., Walling L., Brouquisse R. (2009). Evidence for the existence in *Arabidopsis thaliana* of the proteasome proteolytic pathway: ACTIVATION IN RESPONSE TO CADMIUM. J. Biol. Chem..

[30] Mancosu N., Snyder R.L., Kyriakakis G., Spano D. (2015). Water scarcity and future challenges for food production. Water.

[31] Joshi R., Wani S.H., Singh B., Bohra A., Dar Z.A., Lone A.A., Pareek A., Singla-Pareek S.L. (2016). Transcription factors and plants response to drought stress: Current understanding and future directions. Front. Plant Sci..

[32] Oumarou Abdoulaye A., Lu H., Zhu Y., Alhaj Hamoud Y., Sheteiwy M. (2019). The global trend of the net irrigation water requirement of maize from 1960 to 2050. Climate.

[33] Cañas R.A., Yesbergenova-Cuny Z., Simons M., Chardon F., Armengaud P., Quilleré I., Cukier C., Gibon Y., Limami A.M., Nicolas S. (2017). Exploiting the genetic diversity of maize using a combined metabolomic, enzyme activity profiling, and metabolic modeling approach to link leaf physiology to kernel yield. Plant Cell.

[34] Fallahi M., Saremi H., Javan-Nikkhah M., Somma S., Haidukowski M., Logrieco A.F., Moretti A. (2019). Isolation, Molecular identification and mycotoxin profile of fusarium species isolated from maize kernels in Iran. Toxins.

[35] Liu S., Wang X., Wang H., Xin H., Yang X., Yan J., Li J., Tran L.-S.P., Shinozaki K., Yamaguchi-Shinozaki K. (2013). Genome-wide analysis of *ZmDREB* genes and their association with natural variation in drought tolerance at seedling stage of *Zea mays* L.. PLoS Genet..

[36] Mao H., Wang H., Liu S., Li Z., Yang X., Yan J., Li J., Tran L.-S.P., Qin F. (2015). A transposable element in a *NAC* gene is associated with drought tolerance in maize seedlings. Nat. Commun..

[37] Wang D., Wang L., Su W., Ren Y., You C., Zhang C., Que Y., Su Y. (2020). A class III WRKY transcription factor in sugarcane was involved in biotic and abiotic stress responses. Sci. Rep..

[38] Tunyasuvunakool K., Adler J., Wu Z., Green T., Zielinski M., Zidek A., Bridgland A., Cowie A., Meyer C., Laydon A. (2021). Highly accurate protein structure prediction for the human proteome. Nature.

[39] Guo R., Zhao J., Wang X., Guo C., Li Z., Wang Y., Wang X. (2015). Constitutive expression of a grape aspartic protease gene in transgenic *Arabidopsis* confers osmotic stress tolerance. Plant Cell Tissue Organ Cult. (PCTOC).

[40] Wang X., Wang H., Liu S., Ferjani A., Li J., Yan J., Yang X., Qin F. (2016). Genetic variation in *ZmVPP1* contributes to drought tolerance in maize seedlings. Nat. Genet..

[41] Hajibarat Z., Saidi A. (2022). Senescence-associated proteins and nitrogen remobilization in grain filling under drought stress condition. J. Genet. Eng. Biotechnol..

[42] Sekhon R.S., Saski C., Kumar R., Flinn B.S., Luo F., Beissinger T.M., Ackerman A.J., Breitzman M.W., Bridges W.C., de Leon N. (2019). Integrated genome-scale analysis identifies novel genes and networks underlying senescence in maize. Plant Cell.

[43] Caicedo M., Munaiz E.D., Malvar R.A., Jimenez J.C., Ordas B. (2021). Precision mapping of a maize MAGIC population identified a candidate gene for the senescence-associated physiological traits. Front. Genet..

[44] Chen P., Yan M., Li L., He J., Zhou S., Li Z., Niu C., Bao C., Zhi F., Ma F. (2020). The apple DNA-binding one zinc-finger protein MdDof54 promotes drought resistance. Hortic. Res..

[45] De Caroli M., Lenucci M.S., Manualdi F., Dalessandro G., De Lorenzo G., Piro G. (2015). Molecular dissection of *Phaseolus vulgaris* polygalacturonase-inhibiting protein 2 reveals the presence of hold/release domains affecting protein trafficking toward the cell wall. Front. Plant Sci..

[46] Chen W., Yao Q., Patil G.B., Agarwal G., Deshmukh R.K., Lin L., Wang B., Wang Y., Prince S.J., Song L. (2016). Identification and comparative analysis of differential gene expression in soybean leaf tissue under drought and flooding stress revealed by RNA-Seq. Front. Plant Sci..

[47] Bacete L., Schulz J., Engelsdorf T., Bartosova Z., Vaahtera L., Yan G., Gerhold J.M., Ticha T., Ovstebo C., Gigli-Bisceglia N. (2022). *THESEUS1* modulates cell wall stiffness and abscisic acid production in *Arabidopsis thaliana*. Proc. Natl. Acad. Sci. USA.

[48] Dong Z., Xu Z., Xu L., Galli M., Gallavotti A., Dooner H.K., Chuck G. (2020). *Necrotic upper tips1* mimics heat and drought stress and encodes a protoxylem-specific transcription factor in maize. Proc. Natl. Acad. Sci. USA.

[49] Sun X., Xiong H., Jiang C., Zhang D., Yang Z., Huang Y., Zhu W., Ma S., Duan J., Wang X. (2022). Natural variation of *DROT1* confers drought adaptation in upland rice. Nat. Commun..

[50] Wang N.-N., Xu S.-W., Sun Y.-L., Liu D., Zhou L., Li Y., Li X.-B. (2019). The cotton WRKY transcription factor (GhWRKY33) reduces transgenic *Arabidopsis* resistance to drought stress. Sci. Rep..

[51] Fu J., Wu H., Ma S., Xiang D., Liu R., Xiong L. (2017). OsJAZ1 attenuates drought resistance by regulating JA and ABA signaling in rice. Front. Plant Sci..

[52] Liu Y.-C., Wu Y.-R., Huang X.-H., Sun J., Xie Q. (2011). *AtPUB19*, a U-box E3 ubiquitin ligase, negatively regulates abscisic acid and drought responses in *Arabidopsis thaliana*. Mol. Plant.

[53] Shah T.M., Imran M., Atta B.M., Ashraf M.Y., Hameed A., Waqar I., Shafiq M., Hussain K., Naveed M., Aslam M. (2020). Selection and screening of drought tolerant high yielding chickpea genotypes based on physio-biochemical indices and multi-environmental yield trials. BMC Plant Biol..

[54] Xiang Y., Sun X., Gao S., Qin F., Dai M. (2017). Deletion of an endoplasmic reticulum stress response element in a *ZmPP2C-A* gene facilitates drought tolerance of maize seedlings. Mol. Plant.

[55] Finn R.D., Mistry J., Schuster-Böckler B., Griffiths-Jones S., Hollich V., Lassmann T., Moxon S., Marshall M., Khanna A., Durbin R. (2006). Pfam: Clans, web tools and services. Nucleic Acids Res..

[56] Bailey T.L., Williams N., Misleh C., Li W.W. (2006). MEME: Discovering and analyzing DNA and protein sequence motifs. Nucleic Acids Res..

[57] Guo A.-Y., Zhu Q.-H., Chen X., Luo J.-C. (2007). GSDS: A gene structure display server. Hereditas.

[58] Lescot M., Déhais P., Thijs G., Marchal K., Moreau Y., Van de Peer Y., Rouzé P., Rombauts S. (2002). PlantCARE, a database of plant cis-acting regulatory elements and a portal to tools for in silico analysis of promoter sequences. Nucleic Acids Res..

[59] Ayaz A., Saqib S., Huang H., Zaman W., Lu S., Zhao H. (2021). Genome-wide comparative analysis of long-chain acyl-CoA synthetases (LACSs) gene family: A focus on identification, evolution and expression profiling related to lipid synthesis. Plant Physiol. Biochem..

[60] Walley J.W., Sartor R.C., Shen Z., Schmitz R.J., Wu K.J., Urich M.A., Nery J.R., Smith L.G., Schnable J.C., Ecker J.R. (2016). Integration of omic networks in a developmental atlas of maize. Science.

