# Supplementary material for: Genome-Wide Identification and Analysis of the Maize Serine Peptidase S8 Family Genes in Response to Drought at Seedling Stage
Source: Plants (Basel). 2023 Jan 12;12(2):369. doi: 10.3390/plants12020369 (PMC9865268; doi:10.3390/plants12020369)
Supplement: Supplementary file 1 [file plants-12-00369-s001.zip › Supplementary Figures S1 and S2.pdf]

	Logo	Name	Alt. Name	Width	1.
1.	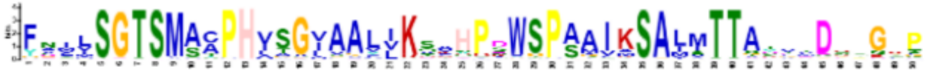	FNILSGTSMACPHVSGVAALIKSAHPDWSPAAIKSALMTTATVTDRSGKP	MEME-1	50	--
2.	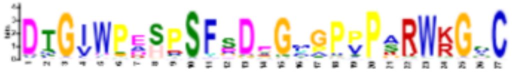	DTGIWPESPSFSDEGMGPPPARWKGSC	MEME-2	27	0.14
3.	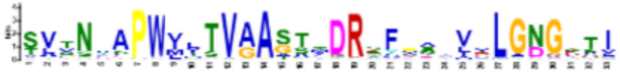	SVTNVAPWVLTVAASTTDRSFPAYVVLGNGQTI	MEME-3	33	0.24
4.	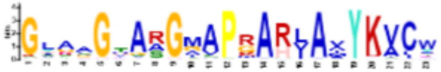	GLAAGTARGMAPRARLAVYKVCW	MEME-4	23	0.18
5.	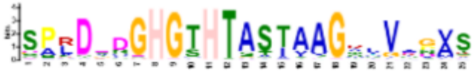	SPRDDDGHGTHTASTAAGRFVAGVS	MEME-5	25	0.21
6.	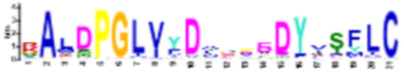	RALDPGLVYDASPEDYVSFLC	MEME-6	21	0.19
7.	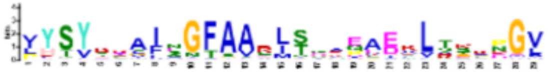	VYSYKHAINGFAARJSNAEAEKLTKRPGV	MEME-7	29	0.2
8.	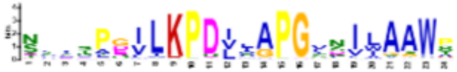	NLVSPGILKPDILAPGVNILAAWP	MEME-8	24	0.21
9.	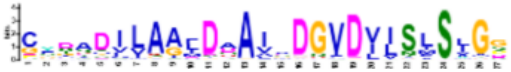	CYDADILAAFDAAIADGVDVJSLSJGG	MEME-9	27	0.18
10.	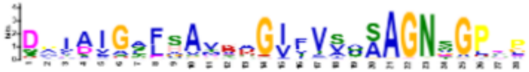	DPIAIGAFSAVKAGIFVSASAGNSGPDP	MEME-10	28	0.23

Figure S1. Schematic of ZmSPS8 conserved motifs. Ten conserved motifs were identified using MEME.

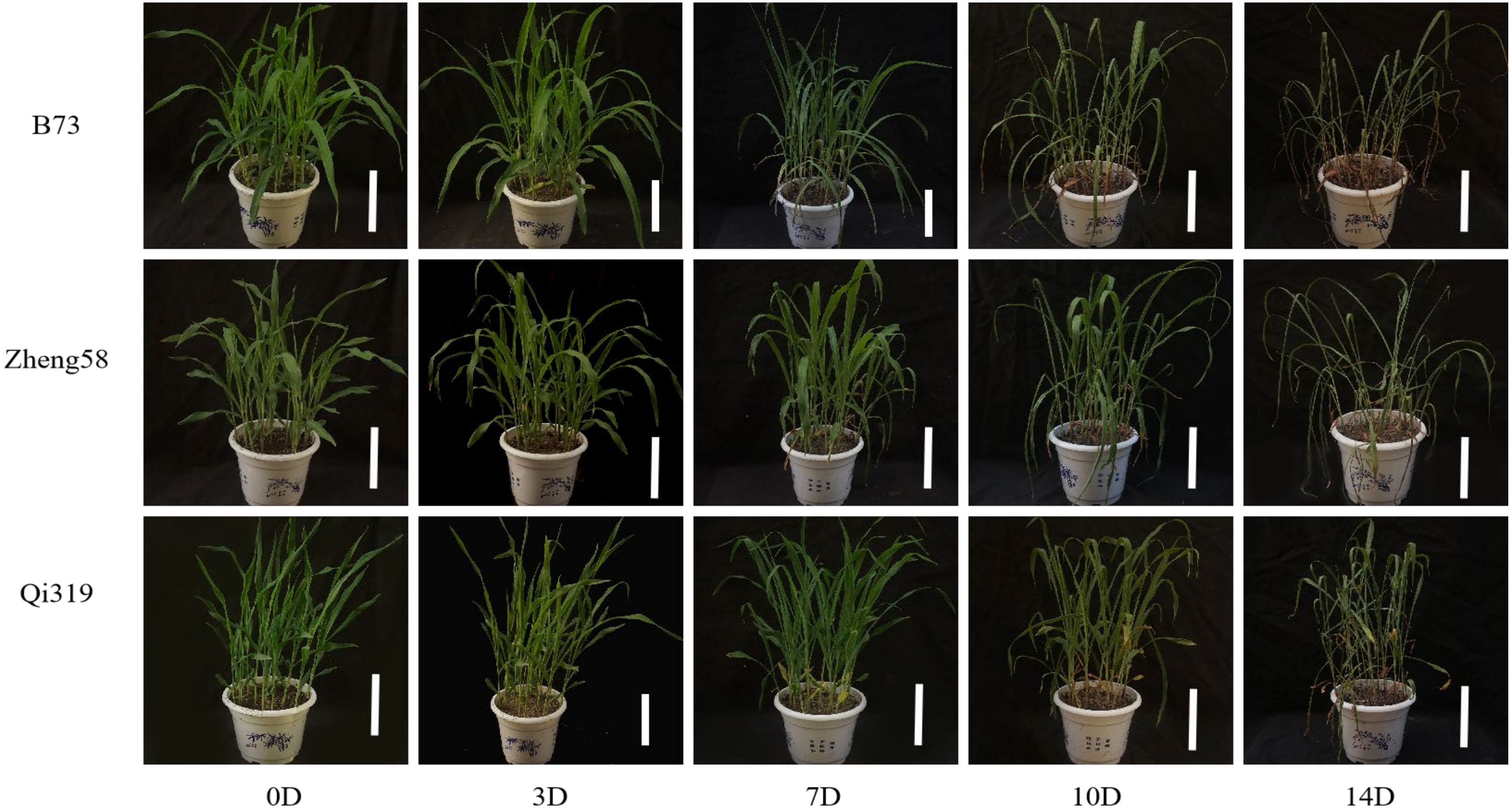

Figure S2. The morphological changes of three maize lines with increasing drought treatment time.
