# Supplementary material for: Genome-Wide Identification and Analysis of the Maize Serine Peptidase S8 Family Genes in Response to Drought at Seedling Stage
Source: Plants (Basel). 2023 Jan 12;12(2):369. doi: 10.3390/plants12020369 (PMC9865268; doi:10.3390/plants12020369)
Supplement: Supplementary file 1 [file plants-12-00369-s001.zip › Supplementary Table S1.pdf]

Table S1. Primer sequences for qRT-PCR analysis

Gene name	Primer sequences
ZmPP2C-A10	F: GTTTCAGCCTGACAGGAAGG R: ACCCTATAGCCGTTCCATTG
ZmSPS8.1.4	F: CGACATCAGCGTTAGAGTCAAG R: CATCCCCCAAGTGTACGATATC
ZmSPS8.1.6	F: GCAAACTGGTCCTCGGCAAC R: GGCTCACACGAGCCGTT
ZmSPS8.1.7	F: CACTGGTATCTTGAAGCCGGA R: GTTAGGATTGTCCGAGGGCG
ZmSPS8.1.8	F: ATCCAAGGAGTACTTACCATGC R: AAAGAGCCAAAAACGTAACCTC
ZmSPS8.1.9	F: ACATGGTGGATGCAAGCTCT R: TGGACCTCTTGAGGAAAAGC
ZmSPS8.1.10	F: TCCGTTCTTGGGAGGTAGCA R: GCTCACGACTCCAGGGTATT
ZmSPS8.1.12	F: GGATCTTGCAATAGAAAGCTCG R: CTTGCATTTCCAAATTGATGCC
ZmSPS8.1.13	F: CAACCAGAGCGAAGTATCAAAC R: CTTGTCATGATGGTGTAGGTCT
ZmSPS8.1.14	F: CCGGTAACTCGTTACTCGG R: CGAAGAGACGACGTAGGAC
ZmSPS8.2.0	F: CTCCTTCTGCTGCTTCGACA R: GACCAAGTACCTCCACTGGG
ZmSPS8.3.1	F: CTTTTGCAAGTTGATAGGGCTT R: GTAGTAGAGATAGGGCGCAATT
ZmSPS8.3.2	F: TGTTGTACAACCATCTGCAAAG R: AGAAGGAGATGACATGGAAGTGC
ZmSPS8.3.3	F: AGGCACATGAAGACATGCAA R: TGTTACCCTCAAGGGTCTGG
